# Racial Disparities in COVID-19-related Deaths in Brazil: Black Lives Matter?

**DOI:** 10.2188/jea.JE20200589

**Published:** 2021-03-05

**Authors:** Paulo Ricardo Martins-Filho, Brenda Carla Lima Araújo, Karyna Batista Sposato, Adriano Antunes de Souza Araújo, Lucindo José Quintans-Júnior, Victor Santana Santos

**Affiliations:** 1 Health Sciences Graduate Program, Federal University of Sergipe, Sergipe, Brazil; 2 Graduate Program in Law, Federal University of Sergipe, Sergipe, Brazil; 3 Consultant, Juvenile Justice and Child Abuse, UNICEF Brazil; 4 Centre for Epidemiology and Public Health, Federal University of Alagoas, Alagoas, Brazil

The Coronavirus Disease 2019 (COVID-19) does not seem to be a democratic disease, and the risk of death has been higher among people living in poor socioeconomic conditions^1^^,^^2^ and among ethnic minorities.^3^^,^^4^ Recent studies published in the *Annals of Epidemiology* have shown how the COVID-19 pandemic has exacerbated racial inequalities and economic marginalization in the United States,^5^^–^^7^ but data from low- and middle-income countries are still scarce. We evaluated the risk of death and the case-fatality rate (CFR) from COVID-19 among Brazilian black people comparing the mortality estimates among the five macro-regions (Northeast, North, Central-West, Southeast, and South) in the country.

To explore racial disparities from COVID-19 in this nationwide register-based study, we extracted information regarding COVID-19 confirmed cases and deaths from the de-identified microdata catalogue and official bulletins for each Brazilian state until August 28, 2020. Black people were defined as individuals who self-identified as African descent. The incidence rate of COVID-19 according to race was estimated using the population estimates provided by the Brazilian Institute of Geography and Statistics (IBGE, acronym in Portuguese). CFR was calculated dividing the number of confirmed deaths from COVID-19 by the number of registered cases for each region. In addition, we estimated the relative risk (RR) and 95% confidence intervals (CIs) of COVID-19 mortality among black Brazilians compared to white Brazilians. Data were analyzed using the statistical software R (R Foundation for Statistical Computing, Vienna, Austria).

Of the 3,002,538 individuals with COVID-19 included in our database, 962,196 (32.0%) were white and 129,541 (4.3%) were black. Despite the incidence rates of COVID-19 were higher among whites, all regions of the country showed higher fatality rates in the black population. Moreover, we found an increased risk of death for blacks compared to whites (RR 1.50; 95% CI, 1.46–1.54; *P* < 0.001) regardless of the Brazilian region (Table 1).

**Table 1.** Incidence rate, case-fatality rate, and relative risk for COVID-19 mortality among black people in Brazil according to macro-regions

Region^a^	White Brazilians				Black Brazilians				Relative risk (95% CI) for COVID-19 mortality among black people	*P*-value
	
	Number of cases	Number of deaths	Incidence rate (per 100,000)	CFR (%)	Number of cases	Number of deaths	Incidence rate (per 100,000)	CFR (%)		
Northeast	161,523	3,998	1,080.7	2.5	47,818	1,733	766.6	3.6	1.46 (1.39 to 1.55)	<0.001
North	29,673	726	799.7	2.5	5,597	167	456.0	3.0	1.22 (1.03 to 1.44)	0.019
Central-West	79,421	1,266	1,302.0	1.6	9,486	255	780.7	2.7	1.69 (1.48 to 1.93)	<0.001
Southeast	588,312	22,603	1,295.5	3.8	61,190	3,915	734.9	6.4	1.67 (1.61 to 1.72)	<0.001
South	103,267	2,619	459.7	2.5	5,450	213	475.5	3.9	1.54 (1.34 to 1.77)	<0.001

Brazil	962,196	31,212	1,038.7	3.2	129,541	6,283	713.6	4.9	1.50 (1.46 to 1.54)	<0.001

^a^Data from seven states (Northeast = 1; North = 3; Central-West = 1; South = 2) were not available and were not included in the results.

CFR, case-fatality rate.

Despite African descents currently representing less than 10% of the Brazilian population, we found a 1.5-fold increased risk for mortality from COVID-19 among black people throughout the national territory, with slight regional differences. These findings may reflect the plethora of regions with different levels of development, social heterogeneity and serious racial problems that remain until nowadays in the Brazilian society and reinforce the misconception of a harmonious multiracial society. Racial disparities in Brazil are strongly associated with socioeconomic environments and precarious conditions of basic sanitation and housing, and therefore these racial disparities have important implications on health outcomes. Furthermore, racial and ethnic minority populations have a disproportionate burden of underlying comorbidities, including hypertension and diabetes, increasing the risk of hospitalizations and deaths related to COVID-19.^8^

The results described in the United States^5^^–^^7^ on racial disparities in the COVID-19 pandemic are similar to those found in Brazil and show that black people in the American continent are at increased risk of exposure and worse outcomes related to COVID-19. High-quality disaggregated data by race and ethnicity are vital to identifying vulnerable populations and planning targeted interventions to reduce disparities on COVID-19 health outcomes.^9^^,^^10^ We assumed that our findings may be biased by the lack of reports from some states and implicit racial bias, but this nationwide study highlights the need of focus attention toward the severity of COVID-19 among black Brazilians.
